# Sociodemographic Disparities in Rectal Cancer Outcomes within Academic Cancer Centers

**DOI:** 10.1245/s10434-025-17085-3

**Published:** 2025-03-02

**Authors:** Susan J. Kim, Chengli Shen, Mohamad El Moheb, Kaelyn C. Cummins, Samantha M. Ruff, Russell Witt, Allan Tsung

**Affiliations:** Department of Surgery, University of Virginia, Charlottesville, VA 22903 USA

## Abstract

**Background:**

Sociodemographic disparities in cancer care outcomes are often related to delayed or limited care access. However, it is unknown whether outcome differences persist after establishing high-level care. This study evaluated the relationship between rectal cancer outcomes and sociodemographic status at academic cancer centers.

**Patients and Methods:**

A retrospective cohort study of patients with rectal cancer treated at academic cancer centers was conducted utilizing the National Cancer Database. Primary outcome was overall survival, while secondary outcomes included 30- and 90-day mortality, time from diagnosis to treatments, hospital readmission rates, and hospital length of stay.

**Results:**

Of the 127,023 patients, median age was 62.7 years (SD 11.92), 59.3% were male, 80.3% were white, and 39.4% presented with stage III disease. After adjustment, Black patients had the worst overall survival (HR 1.10, 95% CI 1.01–1.19, *p* = 0.016). Private insurance status conferred overall survival benefit (HR 0.66, 95% CI 0.58–0.75, *p *< 0.001) as well as the best protection against 30- and 90- day postoperative mortality (30-day OR 0.31, 95% CI 0.10–0.97, *p *= 0.044; 90-day OR 0.37, 95% CI 0.16–0.83, *p *= 0.015). Black patients experienced longer time to first treatment than their white counterparts, with a delay of 3.23 days (95% CI 1.87–4.58, *p *< 0.001).

**Conclusions:**

This study demonstrated the existence of sociodemographic disparities even within the walls of academic institutions, where care should be evidence-based, standardized, comprehensive, and equitable. When analyzing causal pathways, delays in time to treatment initiation may be contributing to these outcomes, but may be modifiable.

**Supplementary Information:**

The online version contains supplementary material available at 10.1245/s10434-025-17085-3.

Disparities exist in all realms of cancer care, including screening,^1,2^ access to care,^3,4^ treatment^5^ and trial participation.^6^ Several studies demonstrate a direct link between sociodemographic factors, delayed access to care, and resultingly worse short- and long-term outcomes, including overall survival.^7–12^ Currently, researchers are evaluating these discrepancies across a multitude of different cancers types^13^ and a spectrum of sociodemographic factors^14,15^ to identify systemic issues and design interventions that improve delivery of cancer care.

Provision of cancer care occurs across a variety of settings, ranging from community-based hospitals to academic institutions, including National Cancer Institute Designated Comprehensive Cancer Centers (CCC).^16^ These facilities meet stringent requirements for external accreditation, have strict regulatory oversight, and employ evidence-based practices to meet designated quality standards.^17^ As a result, academically oriented cancer centers, such as CCCs, care for a high volume of patients with cancer and generally yield superior outcomes.^18–20^

For rare cancers, or those that require significant interdisciplinary management, care at academic facilities is beneficial. Rectal cancer requires extensive multidisciplinary management to coordinate systemic therapy, radiation therapy, and surgery. While there is evidence for improved outcomes at academic compared with community settings,^21^ there is a paucity of information regarding outcome disparities among patients receiving care within the walls of an academic institution once access has been established. Therefore, this study sought to evaluate the association between sociodemographic background and cancer care outcomes for patients with rectal cancer treated at an academic facility.

## Patients and Methods

This study was deemed exempt from informed consent from our institutional review board (IRB) due to the deidentified and publicly available nature of the dataset.

### Patient Selection

**Fig. 1:**
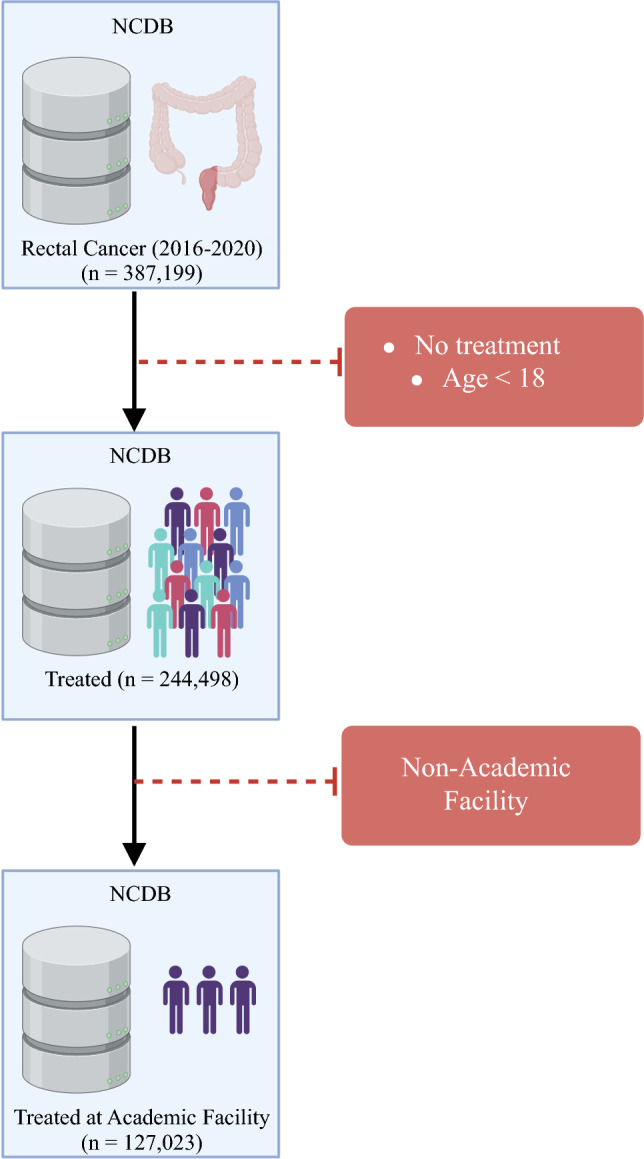
Cohort design

A retrospective study was performed of adult patients from the National Cancer Database (NCDB) from 2016 to 2020. The NCDB is jointly sponsored by the American College of Surgeons and the American Cancer society, and is a registry capturing cancer diagnosis data, patient characteristics, treatment information, and survival outcomes from more than 1500 Commission on Cancer (CoC)-accredited facilities. Patients diagnosed with rectal cancer from 2016 to 2020 were selected from NCDB participant user files using the International Classification of Disease for Oncology, third edition morphology code C19. Of those with a rectal cancer diagnosis, adults over the age of 18 years who received any form of treatment at an academic or CCC-accredited facility were included for our analysis. Academically oriented facilities were identified using NCDB designated codes for Academic/Research Program (including National Cancer Institute-designated comprehensive cancer centers) and Integrated Network Cancer Program. Patients who did not receive treatment were excluded (Fig. 1).

### Patient Characteristics

Demographic and socioeconomic variables were obtained from the NCDB. Self-reported race was categorized into four groups for comparison, and self-reported Hispanic ethnicity was categorized separately. Percentage of individuals without a high school degree was recorded by county and reported as quartiles in the NCDB. We categorized these quartiles into “low,” “moderate,” “intermediate,” and “high” as a surrogate for education status. Median income was categorized into quartiles, similar to education status. Finally, insurance status was categorized into uninsured, private insurance, Medicaid, and Medicare.

American Joint Committee on Cancer (AJCC) staging and Charlson–Deyo comorbidity scores were used to characterize patient clinical characteristics. Treatment-related variables included time from diagnosis to treatment, surgical procedure type, receipt of radiation therapy, and receipt of chemotherapy. Receipt of radiation and chemotherapy specifically were recategorized into groups “not recommended,” “administered as recommended,” “not administered,” and “other.” Surgical procedures were recategorized into “no surgery” and “local” to represent ablative procedures, and “resection” to include full locoregional resection including total mesorectal excision and low anterior resections.

### Outcomes

The primary outcome of interest was overall survival. Secondary outcomes included 30-day and 90-day postoperative mortalities, time from diagnosis to first treatment, length of hospitalization after surgery, and 30-day hospital readmission.

### Statistical Analysis

Descriptive statistics were used to summarize the demographic, clinical, and treatment-related factors of the patient population. The sociodemographic variables of interest included race, ethnicity, income, education, and insurance status. A multivariable Cox regression was conducted to evaluate the independent impact of the aforementioned sociodemographic variables on overall survival. A multivariable linear regression model was constructed to evaluate the independent impact of sociodemographic variables on time to treatment received. Two multivariable logistic regression models were constructed to evaluate the association between sociodemographic factors and 30-day and 90-day mortality. All regression models adjusted for their facility key to address hospital fixed effects, time to from diagnosis to treatment, type of treatment received (chemotherapy, radiation therapy, and surgery), stage, and Charlson–Deyo index. Statistical significance was set at a two-sided *p*-value value of less than 0.05. All statistical analyses were performed using STATA 15.1 (StataCorp LLC), and figures were rendered with R Statistical Software (v4.1.2, R Core Team, 2021).

## Results

### Study Population

A total of 127,023 patients were included in the final analysis, of which 59.3% were male, 80.3% were white, and 92.2% were non-Hispanic. The median age of the cohort was 62.7 ± 11.9 years. The plurality of patients were diagnosed with AJCC stage 3 disease (39.4%), with the remaining stages being relatively balanced. The majority of patients received some form of locoregional treatment (47% resection, 24.5% local), and systemic therapy (64.6%). Baseline characteristics are presented in Table 1.

**Table 1:** Baseline patient characteristics

Factor	Total
	127,023 (%)
*Sex*	
Male	75,307 (59.3%)
Female	51,716 (40.7%)
Age	62.7 (11.92)
*Hispanic ethnicity*	
Non-Hispanic	114,188 (92.2%)
Hispanic	9636 (7.8%)
*Race*	
White	101,940 (80.3%)
Black	15,406 (12.1%)
AAPI	6610 (5.2%)
Other	3067 (2.4%)
*High school education*	
Low	24,123 (22.3%)
Moderate	29,678 (27.4%)
Intermediate	30,389 (28.1%)
High	24,128 (22.3%)
*Median income*	
Low (< $46,277)	19,306 (17.9%)
Moderate ($46,227–57,856)	22,301 (20.6%)
Intermediate ($57,857–74,062)	25,218 (23.4%)
High (≥ $74,063)	41,174 (38.1%)
*Insurance status*	
Not insured	4,925 (3.9%)
Private insurance	56,451 (45.2%)
Medicaid	11,212 (9.0%)
Medicare	50,163 (40.2%)
Other	2016 (1.6%)
Days from diagnosis to treatment	34.381
*Chemotherapy*	
Not recommended	40,941 (32.3%)
Administered	81,777 (64.6%)
Not administered	2656 (2.1%)
Unknown	1251 (1.0%)
*Radiation*	
Not recommended	36,766 (63.7%)
Administered	19,396 (33.6%)
Incomplete	814 (1.4%)
Unknown	751 (1.3%)
*AJCC clinical stage*	
0	745 (2.6%)
1	5335 (18.8%)
2	5509 (19.4%)
3	11,167 (39.4%)
4	5587 (19.7%)
*Charlson–Deyo score*	
0	97,639 (76.9%)
1	20,221 (15.9%)
2	5477 (4.3%)
≥ 3	3686 (2.9%)
*Surgical procedure*	
No surgery	28,980 (28.5%)
Local	24,889 (24.5%)
Resection	47,815 (47.0%)

*AAPI* Asian American and Pacific Islander, *AJCC* American Joint Committee on Cancer

### Diagnosis to Treatment

**Fig. 2:**
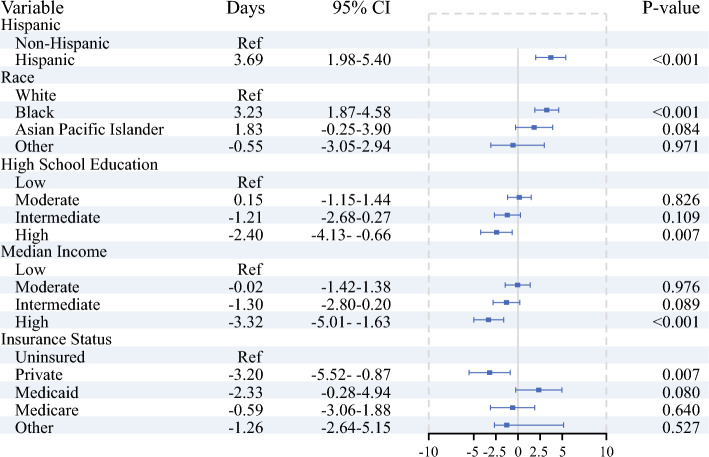
Multivariable linear regression analysis assessing the impact of sociodemographic factors on time from diagnosis to treatment; model was adjusted for facility, chemotherapy and radiation therapy administration, surgery type, AJCC stage, and Charlson–Deyo index

There were significant differences in the time from diagnosis to first treatment based on ethnicity, race, education, income, and insurance status (Fig. 2). On multivariable analysis, Hispanic patients experienced a delay of 3.69 days (95% CI 1.98–5.40, *p* < 0.001) compared with non-Hispanic patients. When compared by race, Black patients had a mean delay to treatment of 3.23 days (95% CI 1.87–4.58, *p* < 0.001) when compared with white patients.

In contrast, higher educational attainment, higher income, and insurance coverage were associated with shorter times to treatment. Patients who lived in an area with high education level received treatment 2.40 days (95% CI −4.13, −0.66, *p* = 0.007) earlier compared with those who lived in areas with low education level. Patients who lived in high-income areas experienced a 3.32-day reduction in time to treatment compared with patients living in low-income areas (95% CI −5.01, −1.63, *p* < 0.001). Insurance status also played a crucial role; private insurance was associated with a 3.20-day reduction (95% CI −5.52, −0.87, *p* = 0.007) in time to treatment compared with uninsured patients.

Location of initial diagnosis was also evaluated. Living in an area of moderate education level made it more likely to receive a diagnosis within an academic facility (OR 0.31, 95% CI 0.08–0.77, *p* = 0.015), with no statistically significant difference found in the income groups (Supplementary Fig. 1). While there was no significant difference based on race or insurance status, the model did exclude categories due to collinearity; therefore, it is difficult to draw conclusions on the impact of either race or Medicaid insurance specifically on facility of diagnosis.

Additionally, this study identified significant differences in time from diagnosis to initiation of first treatment. The time from diagnosis to chemotherapy varied by ethnicity, race, income, and insurance status (Supplementary Fig. 2). Hispanic patients experienced a 5.14-day increase in time to treatment initiation (95% CI 2.76–7.51, *p* < 0.001) compared with non-Hispanic patients, while Black patients experienced a 6.56-day increase (95% CI 4.54–8.57, *p* < 0.001) compared with white patients. Among financial factors, private insurance played a protective role by decreasing time to treatment initiation by 3.87 days (95% CI −7.03, −0.72, *p* = 0.016), as did living in an area with the highest tiers of income, with a 4.22-day decrease (95% CI −6.61, −1.83, *p* = 0.001). Education level did not have an impact on time for diagnosis to chemotherapy initiation. Although there were fewer significant findings in time from diagnosis to radiation, ethnic and racial disparities remained significant. Hispanic patients experienced a 7.72-day increase in time to treatment initiation (95% CI 3.33–12.11, *p* = 0.001) compared with non-Hispanic patients, and Black patients experienced a 5.63-day increase (95% CI 1.95–9.31, *p* = 0.003) compared with white patients (Supplementary Fig. 3).

Regarding time from diagnosis to surgery, we found that Black patients experienced a 5.03-day delay (95% CI 1.72–8.34, *p* = 0.003) compared with white patients, but did not find differences in other races or by ethnicity. Patients with Medicaid for insurance experienced a 10.90-day delay in time to surgery (95% CI 4.19–17.61, *p* = 0.001) compared with the uninsured. There were no differences seen by income or education (Supplementary Fig. 4).

### Treatment-Related Metrics

Length of admission, 30-day readmissions, and 30- and 90-day postoperative mortalities were evaluated as a measure of in-treatment, in-hospital related factors that could contribute to outcome discrepancies. Black patients were found to remain admitted as an inpatient after surgery 0.83 days (95% CI 0.50–1.16, *p* < 0.001) longer than their white counterparts. Significant differences related to insurance were also identified; both private and Medicare insurance holders remained as an inpatient for a shorter duration of time, at 0.91 days (95% CI −1.49, −0.32, *p* = 0.002) and 0.69 days (95% CI −1.32, −0.07,* p* = 0.029), respectively (Supplementary Fig. 5). Despite these differences, there was no difference in 30-day readmission (Supplementary Fig. 6).

**Fig. 3:**
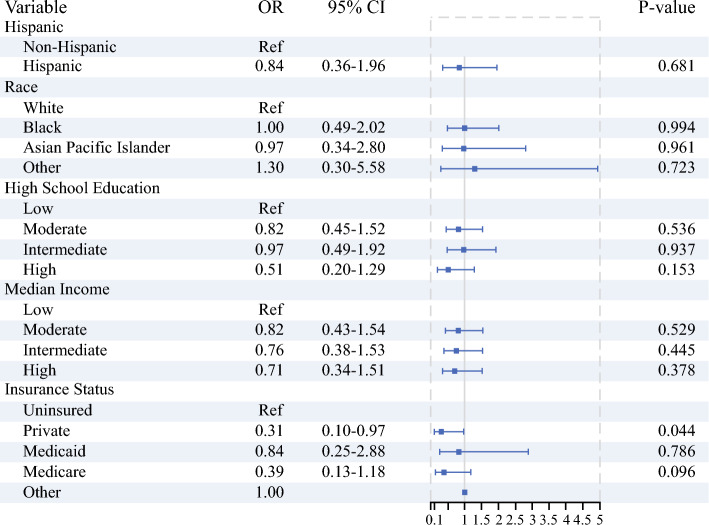
Multivariate logistic regression analysis on the impact of sociodemographic variables on 30-day mortality, adjusted for facility, time from diagnosis to treatment, chemotherapy and radiation therapy administration, surgery type, AJCC stage, and Charlson–Deyo index

**Fig. 4:**
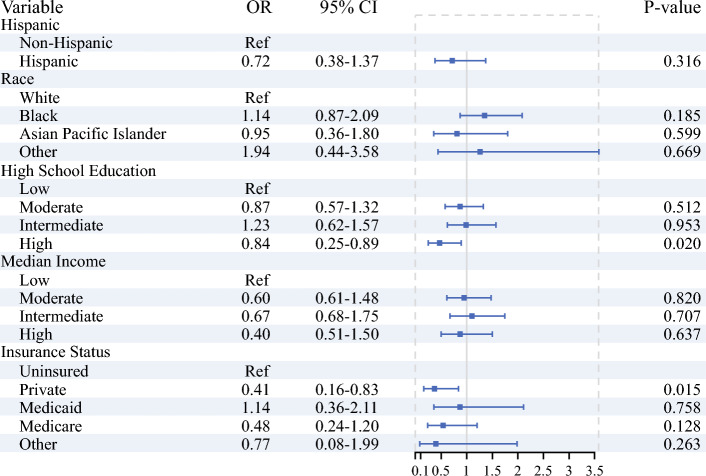
Multivariate logistic regression analysis on the impact of sociodemographic variables on 90-day mortality, adjusted for facility, time from diagnosis to treatment, chemotherapy and radiation therapy administration, surgery type, AJCC stage, and Charlson–Deyo index

For 30- and 90-day postoperative mortality, private insurance was associated with improved short-term outcomes compared with the uninsured (30-day OR 0.31, 95% CI 0.10–0.97, *p* = 0.044; 90-day OR 0.37, 95% CI 0.16–0.83, *p* = 0.015). High education levels were also associated with improved 90-day postoperative mortality (OR 0.47, 95% CI 0.25–0.89, *p* = 0.020), but there were otherwise no significant differences in postoperative mortality across other sociodemographic strata. (Figs. 3, 4).

### Overall Survival

**Fig. 5:**
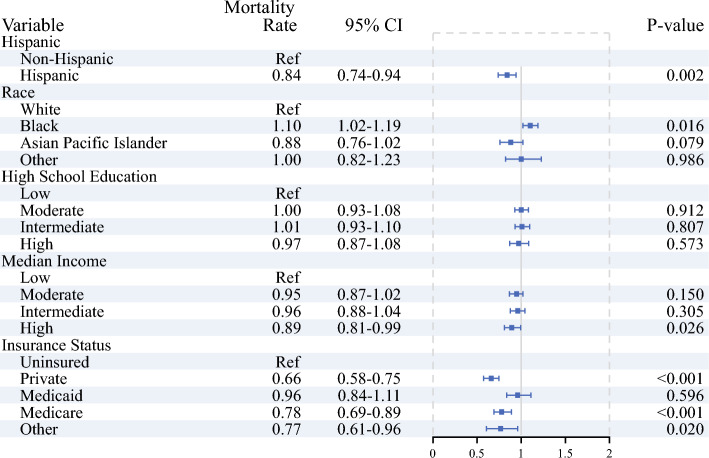
Multivariable Cox regression analysis on the impact of sociodemographic variables on overall survival, adjusting for facility, time from diagnosis to treatment, chemotherapy and radiation therapy administration, surgery type, AJCC stage, and Charlson–Deyo index

**Fig. 6:**
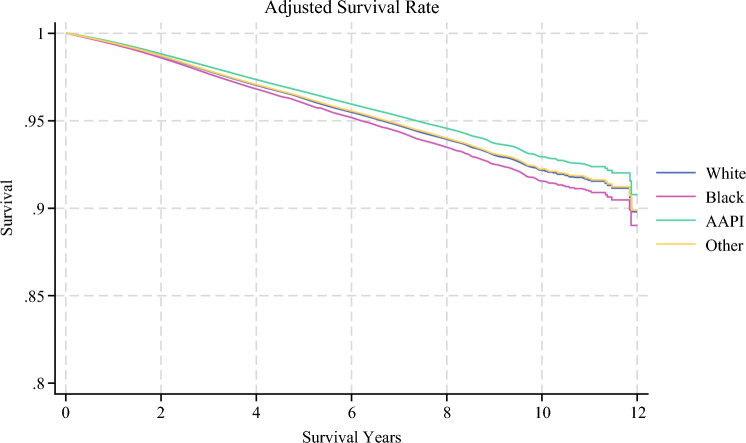
Adjusted overall survival rates by race

After adjusting for clinical characteristics and treatment received, there was a significant difference in overall survival across race, ethnicity, income, and insurance status. Specifically, Black race was predictive of higher mortality rate (HR 1.10, 95% CI 1.01–1.19,* p* = 0.016). In contrast, Hispanic patients (HR 0.84, 95% CI 0.74–0.94, *p* = 0.002), patients living in the area with highest levels of median income (HR 0.89, 95% CI 0.81–0.99, *p* = 0.026), and those with private insurance (HR 0.66, 95% CI 0.58–0.75, *p* < 0.001) or Medicare (HR 0.78, 95%CI 0.69–0.89, *p* < 0.001) experienced a survival benefit (Figs. 5, 6).

## Discussion

Using a large, national, multiracial cohort of patients, this study investigated whether healthcare inequities persisted within academic healthcare environments. While current literature shows that sociodemographic disparities impact cancer outcomes, this has primarily been explored through the lens of access limitation.^22,23^ As such, our study focused on identifying whether sociodemographic differences impact cancer care outcomes among the patients who have established access at an academic facility. This study demonstrates that even after adjusting for potential confounders, sociodemographic disparities in cancer treatment are still present. Differences in race, income status, education level, ethnicity, and insurance status were associated with delays in initiation of treatment, type of therapy received, postoperative outcomes, and overall survival.

Modifiable social determinants of health, such as insurance status, income, and education, influenced outcomes in our study. Private insurance status led to shorter time from diagnosis to treatment, as well as benefits in overall survival. Higher tiers of income mirror this trend. These trends with insurance and income status were consistent with existing literature exploring the impact of these factors on other cancer types. Specifically, those who were underinsured experienced higher mortality rates than their insured or fee-for-service counterparts.^24–28^ We identified that Medicaid recipients are most likely to receive their initial diagnoses at a non-academic facility, suggesting an interplay between poverty and access to high-volume academic facilities, though our analysis did not capture whether this had a consequence on mortality or survival outcomes. Additionally, our study found that private insurance and Medicare status was related to mortality benefit, specifically lower postoperative mortality at 90 days. These findings were not surprising given the extensive resources required for cancer care and postoperative recovery.^29^ Although current literature correlates educational disadvantage with poorer adherence to screening practices,^30,31^ less participation and access to academic care such as clinical trials,^32^ and worse subsequent outcomes,^33,34^ the results of our analysis are more consistent with the few studies demonstrating that education may not be the best socioeconomic predictor of adverse outcomes.^35^

Importantly, we uncovered that Black race conferred worse overall survival despite adjusting for education, income, and insurance status. This is consistent with prior evidence of higher mortality in Black patients that is not fully explained by differences in stage, treatment modality, or insurance status in other cancer types.^24^ When exploring causal factors, we first evaluated the timing between diagnosis and first treatment. We determined where the location of the initial diagnosis occurred, as diagnosis from an outside facility can contribute to delays if testing needs to be repeated or if referral times are lengthy. We did not find that Black patients had an increased likelihood of diagnosis outside of the academic facility where treatment was initiated, but despite this, they still experienced longer times from diagnosis to first treatments, specifically radiation and chemotherapy. Similar to our findings, prior studies have demonstrated higher rates of delayed treatment initiation^36,37^ despite higher odds of later stage diagnosis,^38^ and subsequently worse outcomes in this patient population.^24,25^ Given that the majority of patients in our study had stage III rectal cancer, necessitating upfront chemotherapy/radiation therapy, it is reasonable to conclude this may contribute to the survival differences seen. Interestingly, this pattern was not noted in other races and ethnicities. Hispanic patients were also found to experience longer times to initiate all treatment types, but they were found to have a survival benefit. To be complete, we additionally evaluated for outcomes related to surgical intervention and hospitalization to assess for causal factors of this disparity. There were no variations in 30- or 90- day mortality by race, suggesting surgical intervention itself may not be responsible for these outcome differences. Additionally, we observed no differences in 30- day readmission rates after discharge, suggesting minimal differences in complications related to treatment. What is not captured however, is whether the care received was guideline concordant. With prior studies identifying race as a predictor of receipt of guideline-concordant care, and with only half of non-Hispanic Black patients receiving appropriate treatment,^39^ this is a factor that should be considered.

It is well established that national accreditation of an institution results in improved standards and outcomes. The National Accreditation Program for Rectal Cancer, for example, has resulted in lower risk-adjusted morbidity and mortality after rectal cancer surgery, and NCI CoC accreditation is also associated with outcome improvements.^40^ However, even within academic institutions with accreditation, our study found that there were differences in outcomes and that treatment delays still exist. It is well understood that delays in treatment initiation for a variety of diseases contribute to worse outcomes, including increased mortality, with some studies citing a 6–8% increased risk for colon and rectal cancers for every 4 weeks of diagnostic delay.^41^ Delays in treatment initiation may be attributed to a variety of systemic factors, such as referral patterns, necessity for repeat testing due to outside institution diagnoses, or even scheduling of interventions that are necessary to carry out treatment, such as port placement. Ways to mitigate these processes causing delays in treatment have previously been explored, and may be the key to reversing the outcome discrepancies seen. Particularly in the setting of referrals, other medical subspecialties have used strategies such as high-risk advanced practice provider (APP)-led clinics to determine which patients needed in-personal evaluations to streamline the referral process.^42^ Similarly, the utility of in-person and e-consultations to determine the necessity of in-person provider evaluations has been explored in the setting of cardiology; shifting to e-consultation methods led to a 51.8% reduction in wait time and decreased 1-year incidence rates of all-cause hospitalizations and mortality.^43^ Additional research is required to determine whether measures such as e-visits can mitigate the inequity in care initiation. After establishing the first point of contact, there remains a challenge in initiating necessary steps to facilitate treatment, such as port placement. Optimizing operating room scheduling has been thoroughly explored through several lenses, assessing how to optimize scheduling and operative flow on the basis of case urgency, patient prioritization, and ideal staff allocation. A myriad of tailored strategies have been evaluated, with some hospitals allocating block times for elective cases, scheduling patients into first available spots within 4 weeks then otherwise designating overflow times using explicit prioritization tools with standardized scoring to reduce wait times and provide equitable care.^44^ These measures have had success as high as a 50% mean decrease in wait for day surgeries, and 37% decrease in wait for inpatient operations.^45^

Despite plausible explanations for these outcome disparities, the impact and pervasiveness of systemic racism must be considered when racial differences are noted, despite the difficulty in capturing this phenomenon with data. There is a wealth of literature supporting how institutional policies and unconscious biases contribute to ongoing structural and systemic^46^ racism, disproportionately placing Black and minority patients in socioeconomic disadvantage.^47^ This disadvantage has broad-reaching impacts on both nonmodifiable health risk factors, such as residential segregation leading to increased psychosocial stressors, environmental hazards,^48^ and epigenetic aging,^49,50^ and modifiable adverse health behaviors such as cigarette smoking^51^ and alcohol use.^52^ Racial discrimination has also been correlated with overall inferior care,^53^ including decreased odds of obtaining appropriate screening measures, increased odds of late stage diagnoses,^54^ and increased odds of treatment delay in Black patients.^37^ Even at the foundations of medical innovation, discrimination permeates, with a lack of inclusion in scientific research^55^ and underrepresentation in clinical trial enrollment.^56^ Ultimately, this translates into adverse health outcomes, such as decreased likelihood of achieving textbook postoperative outcomes^58^ and increased mortality,^57^ which are reflected in our findings.

While there are many structural-level changes that can be trialed to improve outcomes, there will always be factors outside of the realm of control. Genomic differences^59–64^ and mutational burden differences between races is a burgeoning area of research, suggesting a biological basis to certain cancer predispositions and outcomes, which may contribute to our findings. Furthermore, epigenetic differences due to the impact of chronic stress^65–70^ and social vulnerability^71–75^ are being examined for similar effect. While these biologic influences are out of our control, improving our understanding of these effects can allow us to systematically implement programs to accommodate for these biological differences and account for their effects, such as resource allocation to socially vulnerable areas to address modifiable social determinants of health.

This study had similar limitations to other studies that address the inequities of health outcomes. Our data are neither comprehensive in terms of the full scope of disparities nor disaggregated in terms of ethnicity or race. The self-reported nature of the database questions impose limitations that prohibit us from encompassing the full spectrum of racial and ethnic diversity. Additionally, we lack the granularity in our data to make concrete associations: county-level high school graduation rates as a proxy for individual educational attainment, reliance on county-level data for income as opposed to individual data, specific causes of treatment delays, inability to identify specific causes of treatment failure or drop out such as partial treatment or transition to hospice care, possible changes in practice, and specific causes of short-term mortality, all of which may influence outcomes.

## Conclusions

In this study, we found that sociodemographic disparities exist for rectal cancer treatment and outcomes, even within the walls of an academic cancer facility. Black patients experienced worse overall survival among patients who have undergone treatment for rectal cancer at an academic cancer-care facility, while the highest quartiles of income and private insurance status conferred a protective benefit. When exploring causative but actionable mechanisms for this disparity, treatment delays were most notable. While the inadequacies and lack of granularity in our current research practice led us to fall short on comprehensive explanations for these obstacles, we must consider the presence of structural and systemic barriers to care that ultimately influence outcomes, and how the healthcare community can change practices to allow comprehensive cancer care to be equitable for all.

## Supplementary Information

Below is the link to the electronic supplementary material.

Supplementary file1 (DOCX 838 KB)
