# Supplementary material for: Sociodemographic Disparities in Rectal Cancer Outcomes within Academic Cancer Centers
Source: Ann Surg Oncol. 2025 Mar 2;32(6):3889–99. doi: 10.1245/s10434-025-17085-3 (PMC12049388; doi:10.1245/s10434-025-17085-3)
Supplement: Supplementary file 1 — Supplementary file1 (DOCX 838 KB) [file 10434_2025_17085_MOESM1_ESM.docx]

**SUPPLEMENTARY MATERIAL**


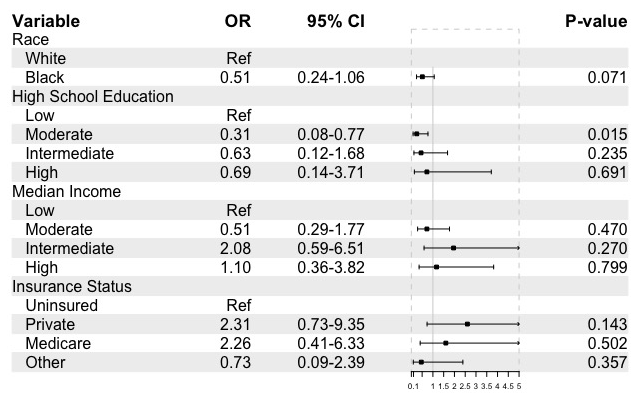


**Supplemental Figure 1.** Multivariable logistic regression analysis assessing impact of sociodemographic factors on diagnosis at versus outside of academic facility. Model was adjusted for facility, chemotherapy and radiation therapy administration, surgery type, AJCC Stage, and Charlson-Deyo Index.


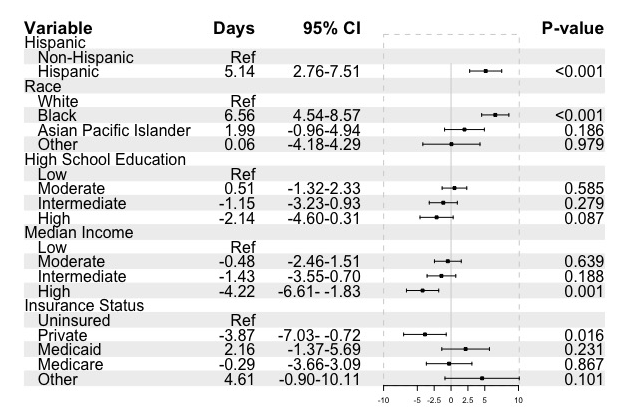
 **Supplemental Figure 2.** Multivariable linear regression analysis assessing impact of sociodemographic factors on time from diagnosis to chemotherapy. Model was adjusted for facility, chemotherapy and radiation therapy administration, surgery type, AJCC Stage, and Charlson-Deyo Index.


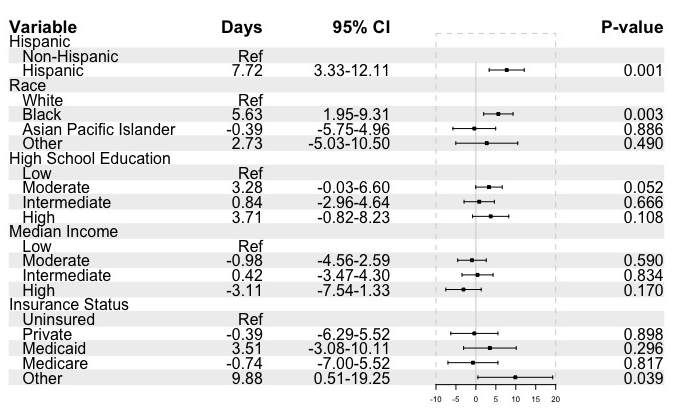


**Supplemental Figure 3.** Multivariable linear regression analysis assessing impact of sociodemographic factors on time from diagnosis to radiation. Model was adjusted for facility type, chemotherapy and radiation therapy administration, surgery type, AJCC Stage, and Charlson-Deyo Index.


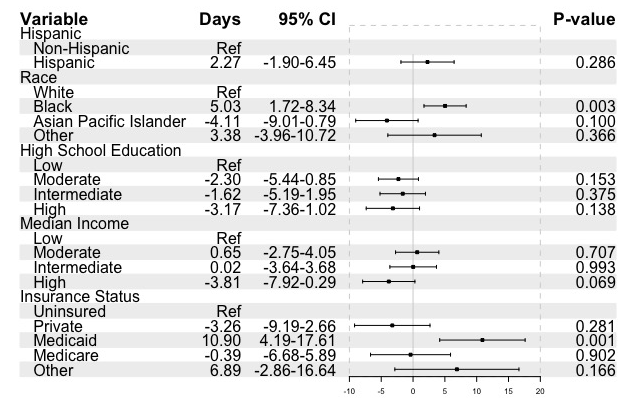


**Supplemental Figure 4.** Multivariable linear regression analysis assessing impact of sociodemographic factors on time from diagnosis to surgery. Model was adjusted for facility type, chemotherapy and radiation therapy administration, surgery type, AJCC Stage, and Charlson-Deyo Index.


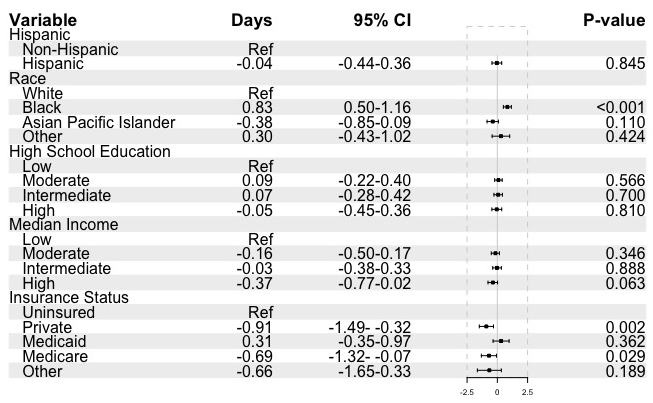


**Supplemental Figure 5.** Multivariable linear regression analysis assessing impact of sociodemographic factors on hospital length stay. Model was adjusted for facility type, time from diagnosis to treatment, chemotherapy and radiation therapy administration, surgery type, AJCC Stage, and Charlson-Deyo Index.


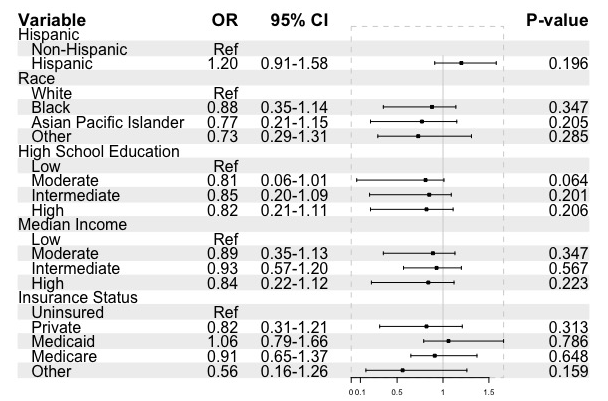


**Supplemental Figure 6.** Multivariable logistic regression analysis assessing impact of sociodemographic factors on 30-day readmission rate. Model was adjusted for facility type, time from diagnosis to treatment, chemotherapy and radiation therapy administration, surgery type, AJCC Stage, and Charlson-Deyo Index.
